# A Scallop IGF Binding Protein Gene: Molecular Characterization and Association of Variants with Growth Traits

**DOI:** 10.1371/journal.pone.0089039

**Published:** 2014-02-19

**Authors:** Liying Feng, Xue Li, Qian Yu, Xianhui Ning, Jinzhuang Dou, Jiajun Zou, Lingling Zhang, Shi Wang, Xiaoli Hu, Zhenmin Bao

**Affiliations:** Key Laboratory of Marine Genetics and Breeding (MGB), Ministry of Education, College of Marine Life Sciences, Ocean University of China, Qingdao, China; Auburn University, United States of America

## Abstract

**Background:**

Scallops represent economically important aquaculture shellfish. The identification of genes and genetic variants related to scallop growth could benefit high-yielding scallop breeding. The insulin-like growth factor (IGF) system is essential for growth and development, with IGF binding proteins (IGFBPs) serving as the major regulators of IGF actions. Although an effect of IGF on growth was detected in bivalve, IGFBP has not been reported, and members of the IGF system have not been characterized in scallop.

**Results:**

We cloned and characterized an *IGFBP* (*PyIGFBP*) gene from the aquaculture bivalve species, Yesso scallop (*Patinopecten yessoensis*, Jay, 1857). Its full-length cDNA sequence was 1,445 bp, with an open reading frame of 378 bp, encoding 125 amino acids, and its genomic sequence was 10,193 bp, consisting of three exons and two introns. The amino acid sequence exhibited the characteristics of IGFBPs, including multiple cysteine residues and relatively conserved motifs in the N-terminal and C-terminal domains. Expression analysis indicated that *PyIGFBP* was expressed in all the tissues and developmental stages examined, with a significantly higher level in the mantle than in other tissues and a significantly higher level in gastrulae and trochophore larvae than in other stages. Furthermore, three single nucleotide polymorphisms (SNPs) were identified in this gene. SNP c.1054A>G was significantly associated with both shell and soft body traits in two populations, with the highest trait values in GG type scallops and lowest in AG type ones.

**Conclusion:**

We cloned and characterized an *IGFBP* gene in a bivalve, and this report also represents the first characterizing an IGF system gene in scallops. A SNP associated with scallop growth for both the shell and soft body was identified in this gene. In addition to providing a candidate marker for scallop breeding, our results also suggest the role of *PyIGFBP* in scallop growth.

## Introduction

Scallops represent an economically important aquaculture species in Asian countries and are consumed worldwide. Among the varieties, the Yesso scallop (*Patinopecten yessoensis*, Jay, 1857) is the main scallop species cultured in Japan and has become one of the most important maricultural shellfish in northern China since it was introduced in 1982 [1]. Similar to other aquaculture species, genetic breeding aiming at improving the growth rate is one of the main focuses of the Yesso scallop farming industry. The identification of genes with a possible function in growth regulation and of genetic markers associated with growth could provide useful information for the genetic improvement of this species.

The insulin-like growth factor (IGF) system which is the important component of the growth hormone axis, plays a pivotal role in cell growth and differentiation [2], [3]. The system mainly includes two IGF ligands (IGF-I and IGF-II, members of a family of insulin related peptides), two IGF receptors (IGF-IR and IGF-IIR), and a family of IGF binding proteins (IGFBPs) [2]. The IGF ligands circulate in the plasma in complexes with IGFBPs with affinities that are equal to or higher than those of IGF-IR, which transports, stores and modulates the bioavailability of IGFs [3]. Therefore, IGFBPs are the major regulators of IGF activity [3]. In addition to modulating IGF bioactivity, the importance of IGFBPs for cell growth has also been indicated in IGF-independent mechanisms [4].

Although most of these studies were implemented in vertebrate species, the IGF system has also been increasingly studied in invertebrates, and its members have been characterized in bivalve species. For example, the insulin-related peptide was identified in *Mytilus edulis* [5], *Crassostrea gigas* [6] and *Anodonta cygnea* [7], and the insulin receptor-related receptor that showed an association with insulin-like effects on growth was found in *C*. *gigas* [8]. Single nucleotide polymorphisms (SNPs) associated with growth were recently identified in the insulin-related peptide gene of *C*. *gigas* [9]. Meanwhile, growth-regulating effects of mammalian IGF on *C*. *gigas* [8] and *Pecten maximus* [10] were also observed. These findings suggest the possible role of the IGF system in the growth regulation of bivalves. However, IGF system members have not been characterized in scallops, and the important regulator of the IGF system, IGFBP, has not been reported in any bivalve species.

In this study, using the transcriptome data of Yesso scallop [11], [12], a cDNA fragment encoding an IGFBP homolog was found. Then, we cloned the full-length cDNA sequence and obtained the genomic DNA sequence of the Yesso scallop *IGFBP* gene (*PyIGFBP*). Its expression levels in adult tissues and developmental stages were characterized. We also identified three SNPs in the transcribed sequence of this gene and found that one of them was significantly associated with the growth of both the shell and soft body of the Yesso scallop. Our data suggest the possible function of PyIGFBP in scallop growth regulation and also provide a candidate locus for the selective breeding of Yesso scallop.

## Materials and Methods

### Sample Collection

Two Yesso scallop populations were collected from Zhangzidao Fishery Group Co., Dalian, the leading producer of Yesso scallop in China. Each population was established by the artificial fertilization of more than 2,000 sexually mature scallops. A total of 60 individuals (13 months old) were randomly sampled in Population I, and 120 individuals (10 months old) were randomly collected from Population II. For all scallops, the shell length (SL), shell height (SH), body weight (BW), soft tissue weight (STW) and adductor muscle weight (AMW) were measured. Tissues including the mantle, gill, gonad, kidney, striated muscle and hepatopancreas were dissected, immediately frozen in liquid nitrogen and stored at −80°C. Embryos and larvae, including newly fertilized eggs, blastulae, gastrulae, trochophore larvae and D-shaped larvae, were also collected and preserved at −80°C.

### RNA Isolation and cDNA Synthesis

Total RNA was isolated from the tissues and embryos/larvae of Yesso scallop using TRIzol reagent (Life Technologies, CA, USA) following the manufacturer’s instructions. The first-strand cDNA was synthesized according to the manufacturer’s instructions using M-MLV Reverse Transcriptase (Promega, WI, USA) in a 25-µL volume with 2 µg of total RNA as the template and 0.8 µM Oligo (dT)_18_ (TaKaRa Biotechnology, Liaoning, China) as the primer. The mixture of total RNA and primer was heated at 95°C for 5 min and then chilled on ice. The first strand cDNA was then synthesized at 42°C for 90 min after adding the reverse transcriptase and its buffer. The cDNA was stored at −20°C and diluted to 1∶40 for use as the template in PCR. To preclude any DNA contamination, a control reaction without reverse transcriptase was performed.

### Full-length cDNA Cloning of *PyIGFBP*

Using the transcriptome data of the Yesso scallop [11], [12], a cDNA fragment of 1,277 bp that encoded an IGFBP homolog was obtained. Then, the 3′ and 5′ rapid amplification of cDNA ends (RACE) were performed to obtain the full-length cDNA from total RNA of mantle tissue from one individual. The reactions were completed according to the instructions of the SMART™ RACE cDNA Amplification Kit (Clontech, CA, USA). Specific primers Igfbp5-f1 and Igfbp5-r1 (Table 1) were designed and used for the 3′ and 5′ RACE, respectively. The RACE products were purified and ligated into the pMD18-T vector (TaKaRa Biotechnology), and six recombinant plasmids from each RACE reaction were sequenced by Sangon Biotech (Shanghai, China). The full-length cDNA sequence was obtained by assembling the sequences of 3′ and 5′ RACE products with the 1,277 bp gene fragment.

**Table 1:** Summary of primers and probes used in this study.

Usage	Locus	Name	Orientation	Sequence (5′ to 3′)
RACE		Igfbp5-f1	Sense	CAGTGTTCCTCACCAATTACATATTTCCCAT
		Igfbp5-r1	Antisense	CGTGTGTCAAACAGGCTAACGAAGTCAT
SNP scanning		Igfbp5-f2	Sense	TCGTTGGACGCTGTGTGTCTCCCT
		Igfbp5-r2	Antisense	TCTAATTACAGTGTCCAGGCCTTAAA
		Igfbp5-f3	Sense	TCCAGTGAGGCAAGTACAACGC
		Igfbp5-r3	Antisense	CGGAAATGCTCACGAGGTCAT
		Igfbp5-f4	Sense	TCTCCATTGCGGTAATATTCCTTATT
		Igfbp5-r4	Antisense	CCAGTGCCAATACATTTTGCG
SNP genotyping	c.-117T>C	Igfbp5sf1	Sense	GGACGCTGTGTGTCTCCCTACT
		Igfbp5sr1	Antisense	TGTCGCTAAACCGATTATTGTCA
		Igfbp5pb1	Probe	CCTGAACGTTGCTT**T**TACGCTACCTAGAA
	c.879C>T	Igfbp5sf2	Sense	TGACTTCGTTAGCCTGTTTGACAC
		Igfbp5sr2	Antisense	TTAGGAATATCTTCTTCTCCCCAACT
		Igfbp5pb2	Probe	GGTTAATCTCAATG**C**AAGTTGGGGAGAAGTT
	c.1054A>G	Igfbp5sf3	Sense	TTGACCTGGACCAGTTCATTCCTC
		Igfbp5sr3	Antisense	TAACACGAAGCAAGCTCTACTATAC
		Igfbp5pb3	Probe	ATTCACTAGAAGAC**G**GTATAGTAGAGCTTGA
qRT-PCR		Igfbp5-f5	Sense	TACCACGGGATCTGCCAGAAT
		Igfbp5-r5	Antisense	GCTGTTGTATGAAGAGTCTACGTGAAG
		HELI-f	Sense	CCAGGAGCAGAGGGAGTTCG
		HELI-r	Antisense	GTCTTACCAGCCCGTCCAGTTC
		UBQ-f	Sense	TCGCTGTAGTCTCCAGGATTGC
		UBQ-r	Antisense	TCGCCACATACCCTCCCAC
		RPL16-f	Sense	CTGCCAGACAGACTGAATGATGCC
		RPL16-r	Antisense	ACGCTCGTCACTGACTTGATAAACCT
		CB-f	Sense	CCTCTCCACCCTTTCTAGTCCTTG
		CB-r	Antisense	CTCCTGGTTCTTCGTCTTTCTCC
		CC-f	Sense	CGTTTTCTCCTGGTTCTTCGTC
		CC-r	Antisense	TCTTCCTCTCCACCCTTTCTAGTC
		His3.3-f	Sense	TAGTATGACTTGCATGATCCGTAGAAA
		His3.3-r	Antisense	GCCAGAAGAATCCGTGGTGAA

The bold and underlined letter in the probe sequence indicates the position of the SNP.

### Sequence Analysis

The assembled cDNA sequence was evaluated by TBLASTX against the National Center for Biotechnology Information database (NCBI, http://www.ncbi.nlm.nih.gov/blast/) for annotation. The genomic sequence of this gene was obtained by searching the genomic data (unpublished) of the Yesso scallop using the full-length cDNA sequence. Then, the genomic and cDNA sequences were compared to obtain the genomic structure of this gene. The deduced amino acid sequence was analyzed using the simple modular architecture research tool (SMART) (http://smart.embl-heidelberg.de/) to predict the conserved domains. The SignalP 4.0 server (http://www.cbs.dtu.dk/services/SignalP/) was used to determine the presence and location of the signal peptide. The ClustalW2 multiple alignment program (http://www.ebi.ac.uk/Tools/msa/clustalw2/) and the GeneDoc multiple alignment editor (http://www.nrbsc.org/gfx/genedoc/index.html) were used to achieve the multiple alignment of PyIGFBP and IGFBP5s from other species.

### Expression Analysis of *PyIGFBP* Gene

The expression levels of *PyIGFBP* in the adult tissues and developmental stages of the Yesso scallop were analyzed using real-time quantitative reverse transcription PCR (qRT-PCR). The first-stand cDNA from adult tissues (mantle, gill, gonad, kidney, striated muscle and hepatopancreas) of 12 Yesso scallops and from embryos and larvae (fertilized egg, blastula, gastrula, trochophore larva and D-shaped larva, n >500, three sets of samples for each stage) was used as the template. For each PCR, three technical repeats were performed. Specific primers Igfbp5-f5 and Igfbp5-r5 (Table 1), which corresponded to the sequences located in exon 2 and exon 3, respectively, were designed for the amplification of the *PyIGFBP* cDNA fragment. Genes encoding DEAD-box RNA helicase-like protein (HELI), ubiquitin (UBQ) and 60S ribosomal protein L16 (RPL16) were selected as reference genes for the tissue samples, and those encoding Cytochrome B (CB), Cytochrome C (CC) and Histone H3.3 (His3.3) were used as reference genes for the embryo/larva samples [13].

The reaction mix included 1× Real-time PCR Master Mix containing SYBR Green dye (TOYOBO, Osaka, Japan), 0.4 µM each primer and 2 µL of the cDNA template. The reaction was performed as follows: initial denaturation at 95°C for 10 min, followed by 40 cycles of 95°C for 15 s and 62.8°C for 1 min. At the end of the PCR, a dissociation (from 95°C to 60°C) analysis was performed to confirm that only one product was amplified. The PCR products for *PyIGFBP* and the reference genes were purified and sequenced by Sangon Biotech to verify the specificity of the qRT-PCR products. All the reactions were performed on a LightCycler 480 system (Roche Applied Science, Penzberg, Germany), and the results were analyzed with Real-time PCR Miner (http://www.miner.ewindup.info/) [14]. The geometric means of the values generated with the three reference genes were calculated for both tissue and embryo/larva samples for normalization [15].

### SNP Scanning and Genotyping

**Figure 1:**
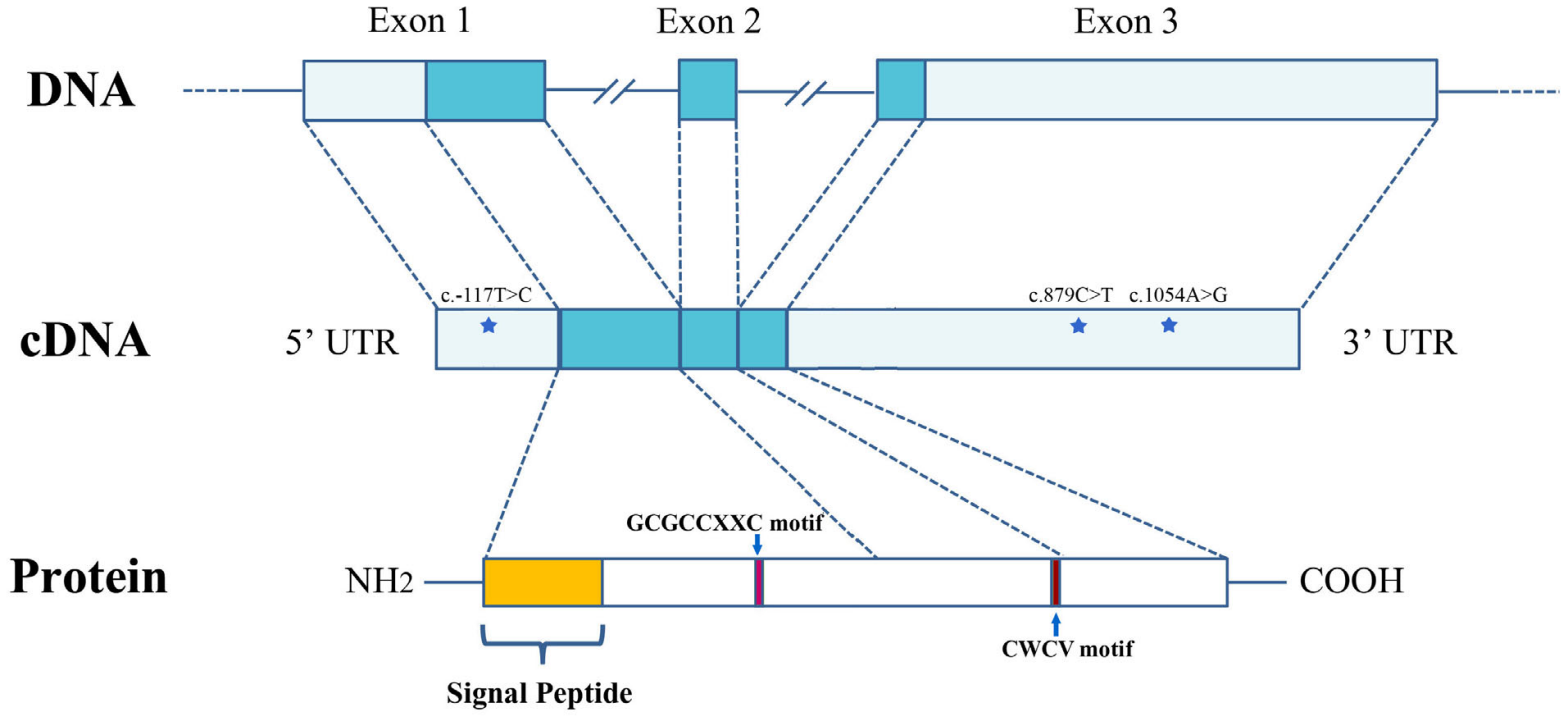
The structure of the *PyIGFBP* gene and protein. The gene contains three exons. The 5′ and 3′ UTR (light blue) and exons (blue) are shown relative to their lengths. The location of the three SNPs (c.-117T>C, c.879C>T and c.1054A>G) is indicated with a star. The position of the GCGCCXXC and CWCV motifs is indicated with an arrow, respectively.

Genomic DNA was extracted from the striated muscle of 180 scallops in the two populations, using the traditional phenol/chloroform extraction method [16]. For SNP screening, the top 5 and bottom 5 scallops in Population I were selected based on the SL values. Three primer pairs, Igfbp5-f2 and Igfbp5-r2, Igfbp5-f3 and Igfbp5-r3 and Igfbp5-f4 and Igfbp5-r4 (Table 1) were used to amplify the DNA fragments that spanned the transcribed sequence of *PyIGFBP*. The 10-µL reaction mix contained 1× Advantege2 PCR buffer, 0.2 mM each dNTP (Life Technologies), 0.2 µM each primer, 50 ng of genomic DNA and 0.2 µL of 50× Advantage2 Polymerase Mix (Clontech). The cycling protocol was the following: 95°C for 5 min; 30 cycles of 95°C for 30 s, 63°C for 30 s and 72°C for 2 min; and a final extension at 72°C for 5 min. Products of the target length were purified by gel extraction and sequenced by Sangon Biotech. Sequences amplified from the same primer pairs were compared among the 10 scallops using the ClustalW2 program. Finally, three candidate SNPs (c.-117T>C, c.879C>T and c.1054A>G) were identified, with one in the 5′ UTR and two in the 3′ UTR (Figure 1).

The three SNPs were then genotyped in the 10 scallops used in the SNP screening by the high-resolution melting (HRM) method for locus verification and marker development [17]. For each SNP, two primers and one probe were designed for the HRM genotyping (Table 1). The 10-µL reaction mix contained 1× PCR buffer, 1.5 mM MgCl_2_, 0.5 U of Taq DNA polymerase (TaKaRa), 0.2 mM each dNTP (Life Technologies), 0.1 µM forward primer, 0.5 µM reverse primer, 1×LCGreen Plus (Idaho Technology, UT, USA) and 20 ng of genomic DNA. The PCR reaction was performed as follows: 95°C for 5 min; 60 cycles of 95°C for 40 s, 63°C for 40 s and 72°C for 40 s; and a final extension at 72°C for 5 min. Then, 3 µl of the probe (10 µM) was added to each reaction mix, and the mixture was denatured at 95°C for 10 min and slowly cooled to 40°C. HRM genotyping was immediately performed using a Light-Scanner instrument (Idaho Technology) with continuous melting curve acquisition (10 acquisitions per °C) over a 0.1°C/s ramp from 40 to 95°C. The data were retrieved and analyzed using the Light Scanner software (Idaho Technology), followed by manually curating the genotype results. All three SNPs were successfully genotyped and further genotyped in other individuals of population I. The SNP that showed a significant association with growth traits in Population I was further genotyped in Population II.

### Statistical Analysis

The scallops from the two populations were grouped according to their genotypes. The chi-squared test was used to examine Hardy-Weinberg equilibrium (HWE) in the populations. For each growth trait, the mean value and standard deviation were calculated for each genotype group. A comparison of the means for each trait among the different genotypes was performed using one way ANOVA with a post-hoc test. The comparison of the expression levels of *PyIGFBP* among adult tissues and among developmental stages was performed using one way ANOVA with a post-hoc test. P values of less than 0.05 were considered statistically significant.

## Results and Discussion

### Characterization of *PyIGFBP* Gene Sequence

Using the transcriptome data of the Yesso scallop [11], [12], a 1,277-bp cDNA fragment of the *PyIGFBP* gene was obtained. After 5′ and 3′ RACE, the full-length sequence of *PyIGFBP* (1,468 bp) was obtained. The gene contained an open reading frame (ORF) of 378 bp (encoding 125 amino acids), a 5′ UTR of 206 bp and a 3′ UTR of 884 bp. A putative polyadenylation signal (AATAAA) was identified at nucleotide positions 1,211 to 1,216. A comparison of the cDNA and genomic DNA sequence of this gene showed that *PyIGFBP* consisted of three exons (405, 97 and 944 bp) and two introns (6465 and 2282 bp), whereas all the identified *IGFBPs* except *IGFBP3* (five exons) in vertebrate species are encoded by four exons [2]. As the genomic information of mollusca *IGFBPs* is currently unavailable in the public database (NCBI), whether the three-exon structure is *PyIGFBP*-specific or common in mollusca species is unknown. All the intron-exon boundaries of *PyIGFBP* conformed to the GT-AG rule [18]. The structure of the *PyIGFBP* gene is shown in Figure 1. The genomic and cDNA sequences of *PyIGFBP* have been deposited in the GenBank database under accession numbers KF801669 and KF801670, respectively.

**Figure 2:**
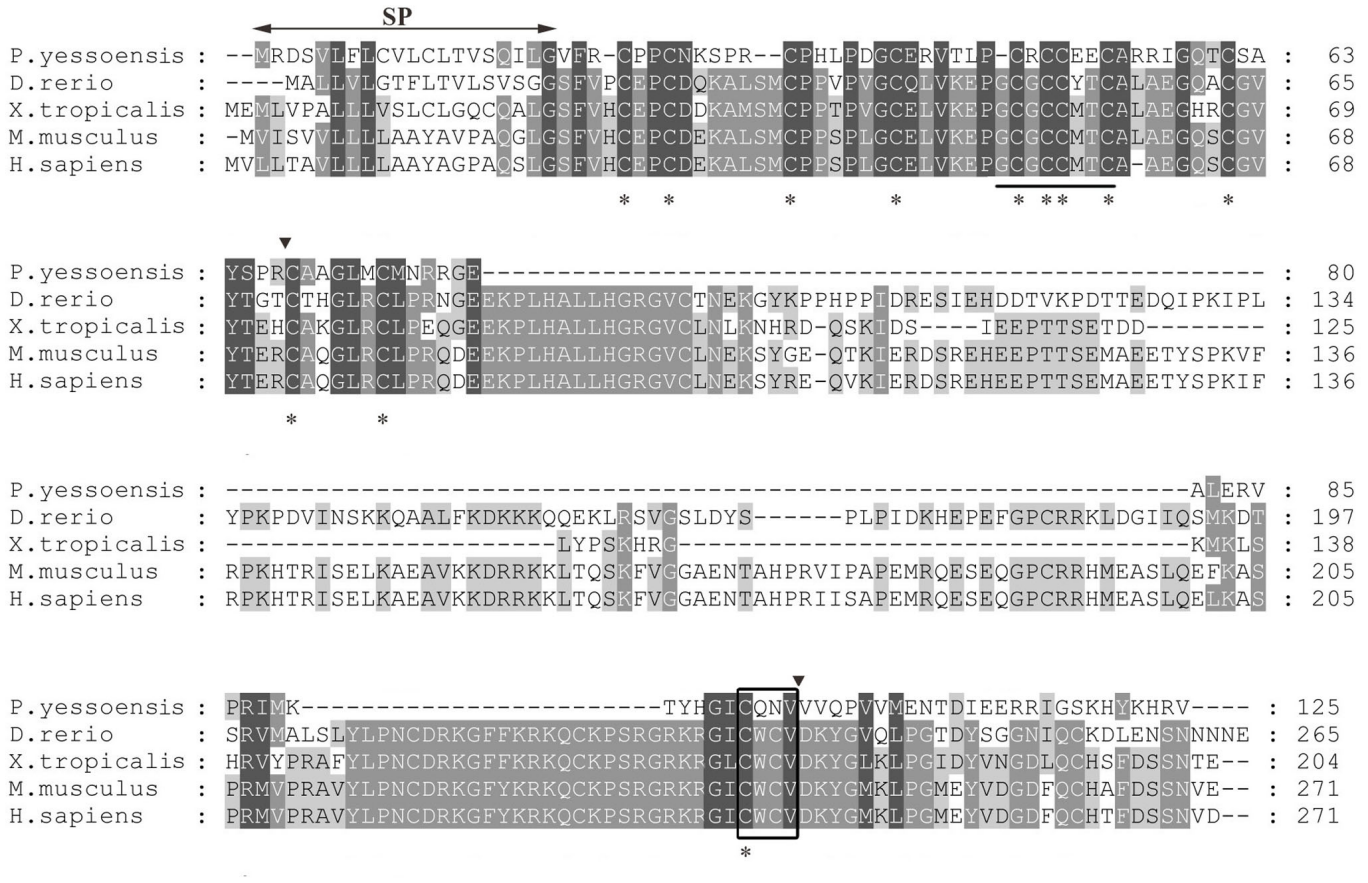
Alignment of the amino acid sequences of PyIGFBP with IGFBP5s from other species. Identical and similar residues are shaded. The putative SP (signal peptide) is indicated with horizontal arrows above the alignment. The characteristic motif of IGFBPs, GCGCCXXC and CWCV, is underlined and marked with a black box, respectively. The 12 conserved cysteine residues are denoted with stars. The black arrowheads mark the intron-exon boundaries in the *PyIGFBP* gene. GenBank accession numbers for IGFBP5s are the following: *D. rerio* (NP_001119935.1); *S. salar* (ABO36535.1); *X. tropicalis* (NP_001016042.1); *M. musculus* (AAH54812.1); *H. sapiens* (CAG33090.1).

TBLASTX analysis showed that the deduced amino acid sequence was most similar to IGFBPs, especially IGFBP5, from other species. To analyze the sequence characteristics of PyIGFBP, the amino acid sequences for PyIGFBP and IGFBP5s in other species were aligned (Figure 2). IGFBPs have been reported to contain conserved cysteine-rich N-terminal and C-terminal domains and a highly variable central linker domain [2]. The alignment showed that there was a long sequence deletion in PyIGFBP in the central region. However, the N-terminal and C-terminal regions were relatively more conserved, which is consistent with the characteristics of IGFBPs. IGFBPs have also been characterized by a conserved GCGCCXXC motif in the N-terminal domain and a CWCV motif in the C-terminal domain, although the significance of these motifs is not yet known [2], [19]. The PyIGFBP exhibited two amino acid differences in the corresponding regions of the GCGCxxC motif (-CRCCXXC) and the CWCV motif (CQNV) (Figure 2). Meanwhile, the number of cysteine residues in the N-terminal and C-terminal regions of vertebrate IGFBP5s is 12 and 6, respectively, and 11 and 1, respectively, in PyIGFBP, mainly due to the deletion of amino acids in the PyIGFBP midregion compared with those from vertebrates. The 20 amino acids in the N-terminal region of PyIGFBP were predicted to be a cleavable signal peptide (Figure 2).

### Spatiotemporal Expression of *PyIGFBP*

**Figure 3:**
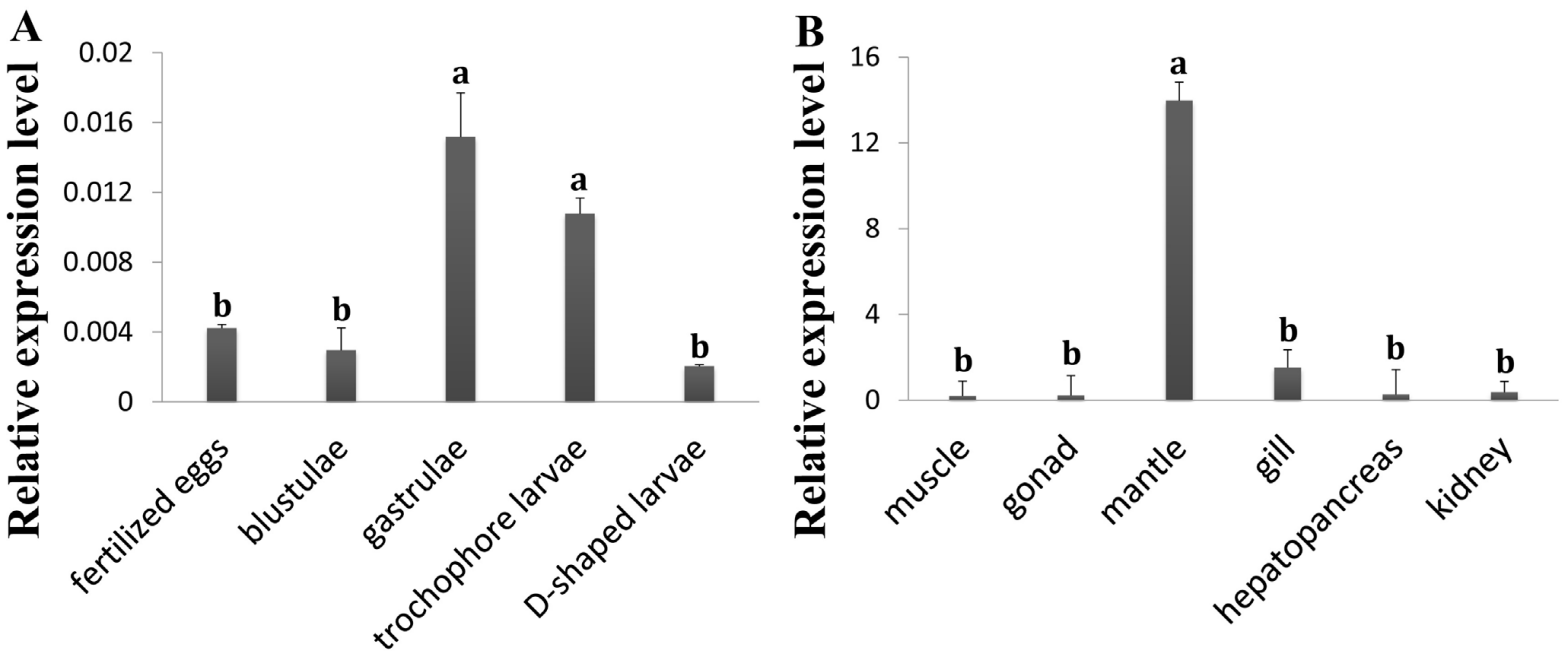
Relative expression levels of *PyIGFBP* in embryos/larvae (A) and adult tissues (B) of the Yesso scallop. Three biological replicates were performed for each developmental stage (n>500 for every replicate), and 12 for each adult tissue. Three technical replicates were conducted for each PCR. The comparison of the expression levels of *PyIGFBP* among different developmental stages and among adult tissues was performed using one way ANOVA with a post-hoc test. Bars with different superscripts indicate significant differences (P<0.05).

The expression of *PyIGFBP* in the embryos/larvae and adult tissues of Yesso scallop was analyzed using qRT-PCR. The amplification efficiencies for *PyIGFBP* and the reference genes were all greater than 0.96 based on the analysis by Real-time PCR Miner. *PyIGFBP* expression was detected in all the developmental stages sampled, including fertilized egg, blastula, gastrula, trochophore larva and D-shaped larva (Figure 3A). *PyIGFBP* mRNA detected in newly fertilized eggs implies that the transcripts might be maternally derived and involved in the early development of scallop embryos. At the gastrula stage, the expression level of *PyIGFBP* increased sharply with a significant difference from that in the fertilized eggs and blastulae. Then, the expression was slightly downregulated in trochophore larvae. In D-shaped larvae, the transcription level of *PyIGFBP* was the lowest among the stages tested. The significantly higher level of *PyIGFBP* mRNA detected in gastrulae and trochophore larvae suggests that the gene might participate in the major organo-morphogenetic events.

*PyIGFBP* expression was also detected in all the adult tissues analyzed (Figure 3B), with a significantly higher level of *PyIGFBP* expression in the mantle, approximately 47, 68, 8, 55 and 30-fold higher than that in striated muscle, gonad, gill, hepatopancreas and kidney, respectively. No significant difference in the *PyIGFBP* expression level was detected among the other five tissues. The mantle is the main tissue in charge of the formation and growth of the shell in bivalves [20], [21]. The calcite and aragonite in the shell are transformed from amorphous calcium carbonate through the regulation by proteins secreted from epithelial cells in the outer mantle tissues [20]–[23]. Many genes expressed in the mantle are responsible for the biomineralization in this process [24]–[27]. The effect of IGF system members on mantle growth and shell formation has been demonstrated in oysters [8]. In mammals, bone is one of the major target tissues of IGF-1 [28]–[30]. IGFBP5 was considered to be the major IGFBP in bone, thereby playing an important role in the modulation of biomineralization [19], [31]. PyIGFBP is more similar to IGFBP5 than to other IGFBP members, as revealed by BLAST analysis. The much higher expression level of *PyIGFBP* in mantle tissue implies its possible role in scallop shell growth.

### Association between *PyIGFBP* SNPs and Growth Traits

We further investigated the sequence variants in *PyIGFBP*. A total of three SNPs, named c.-117T>C, c.879C>T and c.1054A>G, were found in 10 individuals of Population I (Figure 1). SNP c.-117T>C was in the 5′ UTR, and the latter two were in the 3′ UTR. These SNPs were then genotyped in the other scallops of Population I using HRM assays. The genotypes of all the three SNPs were in HWE (Table 2). Potential associations between the genotypes of each locus and each of the five growth traits (SL, SH, BW, STW and AMW) were analyzed *via* one-way ANOVA with a post-hoc test. Significant associations were detected only for one of the three SNPs, c.1054A>G (Table 2). For all of the growth traits measured, the highest and lowest trait values were in scallops with the GG and AG genotypes, respectively, and AA type individuals showed intermediate values. After Bonferroni correction, significant differences in the trait values were detected between GG- and AG-type scallops (p = 0.048 for SL, p = 0.029 for SH, p = 0.048 for BW, p = 0.045 for STW and p = 0.029 for AMW) and between AA- and GG-type ones for STW (p = 0.033).

**Table 2:** Growth traits of Yesso scallops with different genotypes in population I.

Locus	Genotype	N/F	P_HWE_	SL	SH	BW	STW	AMW
c.-117T>C	CC	5/8.9	0.973	57.90±2.79	56.04±2.06	21.14±2.45	11.17±1.61	1.66±0.44
	TT	27/48.2		58.18±5.70	57.74±5.56	22.45±6.23	12.30±4.10	2.06±0.63
	CT	24/42.9		57.78±4.22	57.28±4.21	21.70±4.87	11.81±3.23	1.96±0.45
c.879C>T	TT	18/31.6	0.104	59.24±5.47	59.06±5.55	23.66±5.78	13.05±3.54	2.09±0.62
	CC	16/28.1		57.31±4.04	56.64±4.20	21.23±4.55	11.48±3.22	1.78±0.45
	TC	23/40.4		57.72±4.68	57.22±4.20	21.72±5.44	11.80±3.65	2.01±0.54
c.1054A>G	AA	13/23.6	0.736	57.45±4.94^ab^	56.68±5.14^ab^	20.79±5.47^ab^	11.03±3.57^b^	1.97±0.52^ab^
	GG	13/23.6		61.01±3.83^a^	60.60±3.53^a^	25.57±4.28^a^	14.45±2.82^a^	2.30±0.34^a^
	AG	29/52.7		57.16±4.63^b^	56.63±4.30^b^	21.41±4.88^b^	11.63±3.22^b^	1.85±0.53^b^

N, number of scallops; F, genotype frequency (%); P_HWE_, p value for Hardy-Weinberg equilibrium test; SL, shell length (mm); SH, shell height (mm); BW, body weight (g); STW, soft tissue weight (g); AMW, adductor muscle weight (g). The growth traits are given as the mean ± standard deviation. Within each column of each locus, the value with superscript ^a^ is significantly different from that with superscript ^b^ after Bonferroni correction (P<0.05), and value with superscript ^ab^ is not significantly different from that with ^a^ or with^ b^.

Further evaluation of the association between SNP c.1054A>G and the growth traits in Population II obtained consistent results (Table 3). The highest and lowest trait values were also in GG- and AG-type scallops, respectively. After Bonferroni correction, significant differences were found between GG- and AG-type scallops for traits SL (p = 0.001), SH (p = 0.001), BW (p = 0.003) and STW (p = 0.001), but not for AMW (p = 0.084). Meanwhile, the SH values of AA- and AG-type scallops were significantly different (p = 0.048). The Chi-square test showed that the genotypic frequency at this locus was in HWE.

**Table 3:** Growth traits of Yesso scallops with different genotypes at SNP c.1054A>G in population II.

Genotype	N/F	P_HWE_	SL	SH	BW	STW	AMW
AA	30/25.6	0.890	38.79±5.74^ab^	40.37±5.86^a^	7.45±3.21^ab^	4.25±2.10^ab^	0.80±0.78
GG	29/24.8		42.12±6.29^a^	42.38±6.49^a^	8.58±3.44^a^	4.95±1.89^a^	0.80±0.34
AG	58/49.6		35.91±5.96^b^	37.04±5.77^b^	5.90±3.07^b^	3.29±1.77^ b^	0.55±0.31

N, number of scallops; F, genotype frequency (%); P_HWE_, p value for Hardy-Weinberg equilibrium test; SL, shell length (mm); SH, shell height (mm); BW, body weight (g); STW, soft tissue weight (g); AMW, adductor muscle weight (g). The growth traits are given as the mean ± standard deviation. Within each column, the value with superscript ^a^ is significantly different from that with superscript ^b^ after Bonferroni correction (P<0.05), and value with superscript ^ab^ is not significantly different from that with ^a^ or with^ b^.

Thus, SNP c.1054A>G was significantly associated with four growth traits, including SL, SH, BW and STW, in two populations of Yesso scallop. As the GG-type scallops showed higher trait values than those with the other two genotypes, GG could be the preferred genotype in the selective breeding of Yesso scallops for production improvement. This result also implies the possible role of *PyIGFBP* in the growth regulation of the scallop shell and soft body. Gricourt et al. showed that human recombinant IGF-1 could regulate the shell and soft body growth of oysters and that the insulin-like effects were associated with the expression of oyster insulin receptor-related receptor gene in mantle edge cells [8]. SNP c.1054A>G is located in the 3′ UTR of *PyIGFBP*, and thus this locus might be related to post-transcriptional regulation at the mRNA level or be in tight linkage with the functional variant(s) involved in the regulation of *PyIGFBP* expression and scallop growth. Further in-depth studies focusing on the expression regulation of *PyIGFBP* and its relationship with scallop growth could be helpful to unveil the biological functions of this gene in scallops.
